# Efficacy and Safety of 100 Laparoscopy-Assisted Transgastric Endoscopic Retrograde Cholangiopancreatography Procedures in Patients with Roux-en-Y Gastric Bypass

**DOI:** 10.1007/s11695-020-04946-x

**Published:** 2020-08-23

**Authors:** Lieke M. Koggel, Peter J. Wahab, Rob J. Robijn, Theo J. Aufenacker, Bart P. L. Witteman, Marcel J. M. Groenen, Jan M. Vrolijk

**Affiliations:** 1 Department of Gastroenterology and Hepatology, Rijnstate Hospital, Wagnerlaan 55, 6815 AD Arnhem, The Netherlands; 2 Department of Surgery, Rijnstate Hospital, Arnhem, The Netherlands

**Keywords:** ERCP, Choledocholithiasis, Laparoscopy, Bariatric surgery, Gastric bypass

## Abstract

**Purpose:**

Laparoscopy-assisted transgastric endoscopic retrograde cholangiopancreatography (LAERCP) is an alternative for the anatomically challenging conventional ERCP in patients with a Roux-en-Y gastric bypass (RYGB) as it allows access to the biliary tree via the gastric remnant. We investigated the efficacy and safety of LAERCP.

**Material and Methods:**

We retrospectively reviewed all charts from RYGB patients who underwent a LAERCP between January 2009 and August 2019 in a non-academic referral center for bariatric surgery. Patients who underwent pancreatic therapy were excluded. We collected demographic, clinical, and outcome data. An adverse event was defined as any complaint related to the LAERCP up to 30 days after the procedure and graded according to the ASGE lexicon.

**Results:**

We identified 100 LAERCP in 86 patients with RYGB (70% female, median age 54 years). Same-session cholecystectomy was performed in 35 LAERCP (35%). The papilla of Vater was visualized in 100% of LAERCP with a therapeutic success rate of 94%. Stone extraction succeeded in 88.8% and sphincterotomy was performed in 96.7%. We identified 30 adverse events in 28 procedures, of which eight endoscopy-related, 14 laparoscopy-related, and eight non-specified (f.i. fever, allergic reaction). In total, six severe adverse events were reported concerning post-ERCP pancreatitis (*n* = 2), laparoscopy-related hemorrhage (*n* = 1), abscess (*n* = 1), shock (*n* = 1), and pneumonia (*n* = 1). No patient died due to LAERCP.

**Conclusion:**

LAERCP is an effective and relatively safe procedure for biliary diseases in patients with RYGB.

## Introduction

Obesity is a major and increasing health burden affecting up to 15% of all adults worldwide [1]. Those who are morbidly obese are at greater risk for diseases including diabetes, cardiovascular disease, sleep apnea, gallstones, osteoarthritis, and cancer [1, 2]. Bariatric surgery has been expanding over the last years as it is the most successful option for lasting weight reduction in people who have not had success with diet and exercise. The most performed bariatric surgery in The Netherlands is a laparoscopic Roux-en-Y gastric bypass (RYGB), in which the size of the stomach is reduced (pouch) and a part of the small intestine is bypassed (on average 1/3 of the total length). During weight loss after bariatric surgery, there is an increased risk for the development of gallstones. Total weight loss over 25% after RYGB is associated with gallstone formation [3]. Within 3–12 months after RYGB, cholelithiasis has been reported in 30–47% of patients. Approximately 6–21% of them develop symptoms and require surgical intervention [4–9]. A minority of patients also develop choledocholithiasis.

Endoscopic retrograde cholangiopancreatography (ERCP) is commonly performed in case of biliopancreatic disease, such as choledocholithiasis, cholangitis, and/or biliary pancreatitis. As a consequence of the changed anatomy after RYGB, accessing the papilla of Vater and subsequently the larger bile ducts is a challenge. Conventional (trans-oral) ERCP fails due to the extended route reaching the pancreaticobiliary tract. A variety of techniques have been described in the last decade including EUS-directed transgastric ERCP (EDGE), laparoscopic transcystic common bile duct exploration, endoscopic ultrasound-guided transhepatic ERCP, and percutaneous transhepatic cholangiography (PTC). An endoscopic retrograde approach via the enteroenterostomy is possible, but difficult and with low success rates [10, 11]. Antegrade endoscopic techniques are more successful, of which the laparoscopy-assisted transgastric endoscopic retrograde cholangiopancreatography (LAERCP) technique is most commonly performed.

Although LAERCP is already widely used, only small series are published up until now. In the present study, we describe the efficacy and safety of laparoscopy-assisted transgastric endoscopic retrograde cholangiography (LAERC) in 100 patients with a RYGB performed over the last 10 years in a non-academic referral center for bariatric surgery.

## Patients and Methods

### Patients

We retrospectively reviewed all charts from patients with a RYGB who underwent a LAERCP between January 2009 and August 2019 in Rijnstate Hospital. Patients who underwent pancreatic therapy (cannulation of the ductus pancreaticus for among others pancreatitis, pancreatic cancer, strictures, or leakage) were excluded. Patient characteristics were collected concerning date of birth, gender, BMI, nicotine use, date of gastric bypass, and date of cholecystectomy (if performed). Furthermore, records regarding LAERC and follow-up were gathered. When only the year of gastric bypass surgery or cholecystectomy was known, we used the month July for calculations. When more than one LAERC were performed, we used the date of the first procedure to calculate time difference between RYGB or cholecystectomy and LAERC. Approval from the Medical Ethical Committee was not needed for this type of study.

### Definitions

“Technical success” is defined by visualization of the papilla of Vater. The definition of “therapeutic success” is the completion of intended treatment, such as performance of biliary sphincterotomy, stone extraction, or biliary stent placement. The success rate of stone extraction was noted for all cases in which stones were actually present in the common bile duct during ERC. Performance of a sphincterotomy was noted for all patients in which indicated.

The procedure time was calculated as the time from the first incision to closure, including LAERC/endoscopic procedure and, if indicated, cholecystectomy, adhesiolysis, and/or other surgical procedures. Post-procedure hospital stay refers only to the days of hospital stay after performing the LAERC. Rehospitalization for adverse events is recorded separately.

An adverse event is defined as any complaint related to the LAERC up to 30 days after the procedure. We used the ASGE lexicon for grading of the adverse events [12]. Post-ERCP pancreatitis (PEP) is defined as acute epigastric or left upper quadrant abdominal pain associated with an increase in lipase three times the upper limit of normal or associated findings on a CT scan (when performed). As CRP is always elevated shortly after the procedure because of the laparoscopy, we measured the severity of the pancreatitis by pain presentation, duration of hospitalization, and follow-up of CRP. When it was unclear if an adverse event was endoscopy- or surgery-related, such as fever of unknown origin, atrial fibrillation, or allergic reactions, they were classified as non-specified.

### Statistics

Descriptive statistics (median and range) were used to present variables of interest. Differences in adverse event rate between two groups were measured by using the X2 test. A *p* value less than 0.05 was considered to be significant. Statistical analyses were performed using SPSS version 25.0 (SPSS, Inc., Chicago, IL).

### Procedure

**Fig. 1:**
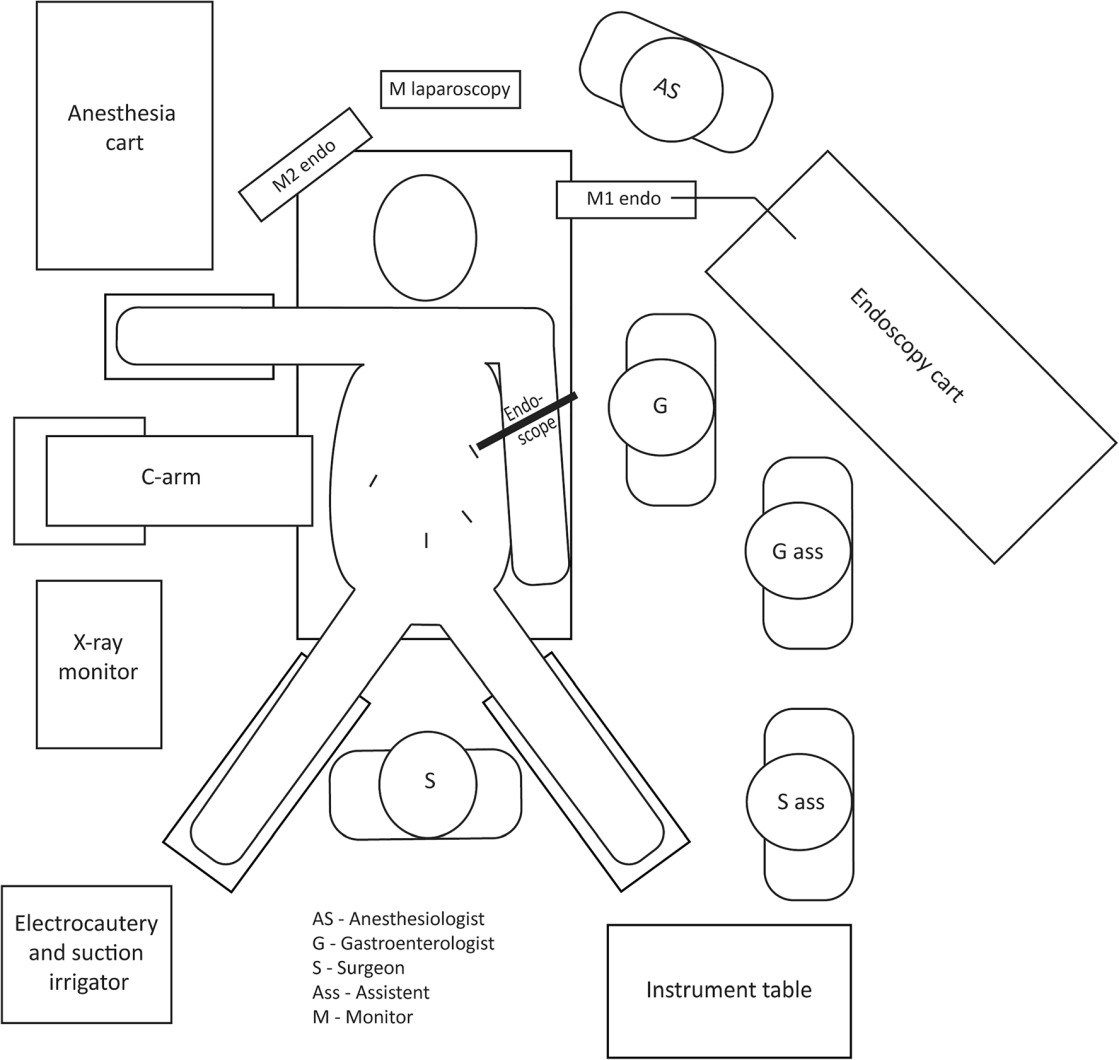
Position in operation room

See Fig. 1 for the operation room setup. The patient was positioned in supine split-leg position (French position), prepped, and draped in the conventional manner. All LAERC were performed under general anesthesia with antibiotic prophylaxis. All patients received diclofenac 100 mg rectal or intravenous as PEP prophylaxis. Pneumoperitoneum was achieved through a standard Palmer’s point entry port. A 15-mm trocar was placed in the left hypochondrium. If necessary, adhesions were lysed to identify the remnant stomach. A gastrostomy was made diathermically in the gastric remnant at about 18-cm distance from the pylorus. The trocar was placed inside the gastric remnant at an angle supportive of introduction of the scope and the position was stabilized by purse string sutures. Before the unsterile duodenoscope entered the gastric remnant, the trocar was covered with a sterile drape with opening. The ERC was performed in a conventional manner by the gastroenterologist through this trocar. Afterwards, the gastrostomy was closed with a V-Loc (Medtronic, Minneapolis, MN, USA) suture. If indicated, a cholecystectomy was performed before or after the ERC. The laparoscopy was completed in a conventional manner. All laparoscopies were performed by surgeons with high-volume laparoscopic experience. All ERCs were performed by one of four endoscopists with high-volume ERCP practices (average > 75 ERCP/year, total > 750 ERCPs/endoscopist).

## Results

### Patient Data

We identified 100 LAERC in 86 patients with RYGB of whom 70% female. Eight patients underwent a second LAERC and three patients needed a third LAERC. Patient descriptives are shown in Table 1. The median age at LAERC was 54 years. LAERC was performed at a median time of 27 months after RYGB (exact month unknown in 4 patients). Median total weight loss since RYGB was 32% (− 20–51%) of the initial body weight. Three patients gained weight since the gastric bypass operation. All three patients underwent a revision to a RYGB secondary to a sleeve gastrectomy or Mason-MacLean procedure. In 46 patients (53%), cholecystectomy was performed on a separate prior occasion. Median time between cholecystectomy and LAERC was 6 months (exact month unknown in 13 patients).

**Table 1:** Patient descriptives (*n* = 86)

Median age, years (range)	53.5 (27–72)
Female, no (%)	70 (81.4)
Smoking status, no (%)	
Current smoker Former smoker No smoker Unknown	13 (15.1) 7 (8.1) 55 (64.0) 11 (12.8)
Median body mass index, kg/m^2^ (range)	29.32 (20.06–49.84)
Median weight loss since RYGB, % (range)	31.89 (− 20.00–50.78)
Median time since RYGB, months (range)	27 (0–177)
Cholecystectomy prior to LAERC, no (%) Median time since cholecystectomy, months (range)	46 (53.5) 6 (0–211)

### Procedure

Table 2 shows the procedural data. LAERCs were performed in an acute setting in 55% and elective in 45% of cases. Thirty-five patients (41%) underwent cholecystectomy in the same setting. Half of these same-setting cholecystectomies were performed previous to ERC (47%). In five patients, cholecystectomy was not performed during LAERC because of a failed ERC (*n* = 2), severe pancreatitis (*n* = 1), ongoing cholangitis (*n* = 1), or perioperative diagnosis of metastatic cancer (*n* = 1).

**Table 2:** Procedural data (*n* = 100)

ERC in acute setting, no (%)	55 (55.0)
Indication, no (%)	
Choledocholithiasis Cholangitis CBD stent placement for cystic duct leakage Stent removal Other	61 (61.0) 25 (25.0) 7 (7.0) 5 (5.0) 2 (2.0)
Success rate, no (%)	
Technical success Therapeutic success Cannulation CBD Stone extraction (*n* = 54) Sphincterotomy (*n* = 92)	100 (100) 94 (94.0) 95 (95.0) 48 (88.8) 89 (96.7)
Median total procedure duration, min (range)	1:20 (0:35–3:30)
Without cholecystectomy (*n* = 64) With cholecystectomy (*n* = 36)	1:11 (0:35–2:54) 1:47 (0:39–3:30)
Median hospital stay, days (range)	2 (1–14)

Median procedure time was 1 h and 20 min (0:35–3:30). The difference in time of procedure with or without cholecystectomy was not statistically significant (*p* = 0.296). When re-LAERC was expected on short notice for among others CBD stent removal, a percutaneous endoscopic gastrostomy (PEG) tube was placed in the remnant stomach during procedure (*n* = 6). Other reasons for prolonged durations were correction of internal herniation (*n* = 8), perioperative bleeding (*n* = 7) and adhesiolysis (*n* = 6). In two patients, correction of internal herniation occurred as well as adhesiolysis or PEG tube placement. Two LAERC lasted more than three hours: One due to both difficult CBD cannulation as of difficult cholecystectomy due to adhesions and the other due to a failed ERC followed by percutaneous transhepatic biliary drainage and rendezvous procedure. When not prolonged and without cholecystectomy, the median total procedure time was 1 h and 4 min (0:35–2:23).

Most of the patients were discharged after a median hospital stay of 2 days after LAERC. In total, 30 patients were admitted for an extended period up to 14 days. In 14 cases because of LAERC-associated adverse events. One patient suffered from both bleeding due to sphincterotomy and bile duct leakage due to surgery. Another patient complained of abdominal pain which could be related to the procedure as well as obstipation. In the remaining 14 patients, the prolonged hospitalization was not related to the LAERC.

### Indication and Efficacy

The majority of LAERC were performed because of choledocholithiasis (*n* = 61) or cholangitis (*n* = 25). In seven cases, a stent was placed in the biliary duct, all because of remnant cystic bile duct leakage shortly after prior cholecystectomy. Two patients underwent LAERC because of presumed sphincter of Oddi dysfunction. One of them was relieved of complaints after sphincterotomy. In the other patient, cannulation of the common bile duct failed. Overall, the technical success rate of LAERC was 100% with completion of the intended treatment in 94% of procedures. Stone extraction succeeded in 88.8% and a sphincterotomy was performed in 96.7%. In case stone extraction failed, patients underwent a successful second LAERC (*n* = 2, whereof one rendezvous procedure) or ERC via PEG tube in the remnant stomach (*n* = 1), stones were not present anymore during second LAERC (*n* = 1), or symptoms resolved without the need of further interventions (*n* = 2).

### Adverse Events

In total, 30 adverse events were reported concerning 28 procedures as shown in Table 3. Six of them were scored as severe, concerning PEP (*n* = 2), laparoscopy-related hemorrhage (*n* = 1), laparoscopy-related abscess/infected hematoma (*n* = 1), shock (*n* = 1), and pneumonia (*n* = 1). The other adverse events were graded as moderate (*n* = 10) or mild (*n* = 14).

**Table 3:** Adverse events

	Overall	Severe*
ERC-related, no (%)	8 (8)	2 (2)
Post-ERCP pancreatitis Hemorrhage Perforation	4 (4) 3 (3) 1 (1)	2 (2) - -
Laparoscopy-related, no (%)	14 (14)	2 (2)
Hemorrhage Wound infection ACNES** Other	5 (5) 3 (3) 2 (2) 4 (4)	1 (1) - - 1 (1)
Non-specified, no (%)	8 (8)	2 (2)

*According to ASGE lexicon, **Anterior cutaneous nerve entrapment syndrome

Eight adverse events were endoscopy-related: four PEP, three papillary hemorrhages, and one perforation. The PEP patients included three females (28, 54, and 54 years) and one male (45 years). In two of them, the pancreatic duct had been cannulated unintentionally during the procedure. One received a temporary pancreatic duct stent. Two of the four cases were interpreted as severe: One because of intense pain, hospitalization for 11 days, and a CRP elevation in the days after laparoscopy till 234 mg/L; and the other because of a re-laparoscopy that was performed for presumed perforation due to intense pain, which proved to be not the case. The other two were graded as moderate and showed quick relief of pain. One hemorrhage occurred after placement of a plastic endoprosthesis and stopped spontaneously. The other two hemorrhages occurred after restarting anticoagulants. One patient received packed cells and had to stop the anticoagulant for a longer period of time. In the other patient, a reversal agent was used to stop the bleeding. The perforation occurred during a difficult LAERC with fausse route and had no clinical consequences.

Fourteen adverse events were laparoscopy-related: five hemorrhages, three wound infections, two anterior cutaneous nerve entrapment syndrome (ACNES), one fluid collection, one abscess/infected hematoma, one case of fever due to gastrostomy tract leak, and one case with subcutaneous emphysema after intubation. All the hemorrhages occurred in procedures with same-setting cholecystectomy. One was scored as severe due to the need of a re-laparoscopy and temporary stay at the intensive care unit. The other four hemorrhages were adequately treated perioperative and had no further clinical consequences. The abscess/infected hematoma also occurred in a procedure with same-setting cholecystectomy and was scored as severe because the patient had been re-admitted twice regarding this adverse event. The overall adverse event rate in patients who underwent a LAERC combined with a cholecystectomy was 33.3%. The adverse event rate for LAERC without same-setting cholecystectomy was 25% (*p* = 0.487).

Two severe non-specified adverse events were seen. One patient ended in shock, was admitted to the medium care unit for 4 days, and underwent a re-laparoscopy. Another patient suffered pneumonia wherefore 14 days of hospitalization. Furthermore, pneumonia (*n* = 3 including the severe pneumonia), *E. coli* bacteremia (*n* = 1), atrium fibrillation (*n* = 1), asystole post-surgery solved by means of a precordial thump (*n* = 1), and exanthema due to an allergic reaction (*n* = 1) were reported.

None of the procedures had to be converted to an open procedure. No mortalities were reported at all.

## Discussion

We report the results of the largest series LAERC to date. When performed by experienced laparoscopic surgeons and endoscopists, LAERC showed high therapeutic success rates (94%). Adverse event rate was high (28%). However, as these patients are more at risk for adverse events due to concomitant cholecystectomy, prior surgery, and/or obesity, we consider LAERC as relatively safe. Most adverse events were caused by surgical difficulties regarding adhesions and bleeding (47%). ERC-related adverse events were comparable with regular ERCP procedures [13].

We encountered a number of difficulties with regard to the procedure. First, the logistics can be an obstacle when planning a LAERC due to multiple specialties involved. In our experience, this can be solved by clear communication and agreements regarding responsibility, finance, and by dedicated planners and/or involved doctors. Secondly, there are multiple challenges compared with “conventional ERC” to overcome: the patient is positioned in supine position instead of prone position which influences endoscopic orientation and fluoroscopic images and increases the chance of unintentional introduction of the ductus pancreaticus. The X-ray permeability of most operation room tables is less than the tables we use for conventional ERCs. Due to several surgical instruments during the procedure, the X-ray image is disturbed. The presence of equipment for both the laparoscopy as well as the endoscopy procedure makes most operation rooms overcrowded. We have chosen for the setup as presented in Fig. 1, increasing the distance to the X-ray monitor. Access of the endoscope through a trocar through the abdominal wall results in an endoscopic position similar to the “long position” in conventional ERCP. In this position, the distance from the tip of the endoscope to the papilla was wider and cannulation more difficult. This can be overcome by positioning the trocar as far as possible in the left upper hypochondrium and pointing diagonally through the gastric remnant allowing a straight access to the pylorus. However, adhesions or changed bowel positions after RYGB can complicate an ideal trocar position. In addition, the access from the trocar to the remnant stomach must be properly sealed around the 15-mm trocar (purse string suture) or fixated to the stomach with surgical instruments. Otherwise, there is too much leakage of insufflated CO_2_ so that the stomach and duodenum do not unfold and of course contamination of the abdominal cavity.

When looking at adverse events, diagnosing a PEP and its severity is more of a challenge because of abdominal pain and an already elevated CRP due to laparoscopy. For diagnosing PEP, pain in combination with an elevated lipase can be conclusive as lipase does not increase due to a laparoscopy. Follow-up of the CRP over time and close pain observation can help to predict the severity. Although non-steroidal anti-inflammatory drugs (NSAIDs) are relatively contraindicated in RYGB patients, single admission prior to LAERC to prevent a PEP appears to be safe. Additionally, a preventive pancreatic duct stent placement after unintentionally cannulation of the pancreatic duct is still possible, as this stent will normally fall out within a few days and does not require a re-LAERCP.

Previous studies already showed high cannulation rates of transgastric ERCP up to 96–99%, conforming with our results [10, 14, 15]. Notable is that our overall adverse event rate of 28% is higher than earlier described adverse event rates (14–18%) [10, 14, 15]. Possibly because of the high percentage of patients that underwent a same-setting laparoscopic cholecystectomy which entails an additional risk of adverse events. A study in which 39% of LAERCP were combined with a cholecystectomy also showed a higher adverse event rate of 35.4% [16]. Besides, a higher percentage of laparoscopy-related adverse events was seen in our group compared with previous studies (14% vs. 8–11%) [10, 14, 15]. The 8% ERC-related adverse events are comparable with earlier described ERCP-related adverse events of transgastric ERCP (6–7%) and the adverse event rate of conventional ERCP in general (7%) [10, 13–16]. As the endoscope is unsterile, this may have contributed to the 3% wound infections that occurred despite antibiotic prophylaxis as they generally occur in only 0.3% of laparoscopies [17]. Conversion rates to open procedure up to 8% have been described, mostly because the pylorus was difficult to access due to inconvenient placement of the gastrostomy tube in the gastric remnant [15]. None of our procedures had to be converted. This difference can possibly be explained by distinction in experience of the specialists involved.

Single balloon- or double balloon-assisted enteroscopy ERCP showed lower adverse event rates than LAERCP (8–10% and 2–10% respectively). However, it is questionable if this outweighs the moderate success rates (62–75% and 73–82% respectively) [10, 11]. As success rates are associated with the length of the Roux limb + ligament of Treitz to jejunojejunal anastomosis limb, it is suggested that balloon-assisted enteroscopy ERCP should only be considered in case of a length of less than 150 cm [18]. In case of PTC, an interventional radiologist gains percutaneous access to the intrahepatic biliary system. PTC showed high success rates of approximately 90% for removal of biliary stones, can be performed under local anesthetics, and is less invasive than LAERCP [19, 20]. However, adverse events such as hemorrhage, cholangitis, and bacteremia are seen in around 20% and the procedure can be burdensome [21]. Less commonly performed is the EDGE, a procedure in which a lumen-apposing metal stent is placed using EUS to access the gastric remnant. EDGE showed similar success (97–100%) and adverse event (20–24%) rates in comparison with LAERCP [22, 23]. Looking at procedure time and hospital stay, EDGE favors LAERCP [22]. However, we have a deep concern regarding gastro-gastric fistula as an adverse event of EDGE. Also, as the stent needs some time to unfold, the ERCP cannot be performed directly afterwards which makes this method only suitable for staged settings and not for acute settings, which includes 55% of our procedures.

The laparoscopic approach of the ERC benefits the possibility of performing a cholecystectomy in the same setting. If indicated, the cholecystectomy and/or correction of internal herniation are preferably performed previous to the ERC. This is because of bowel inflation during the endoscopic procedure which can limit the surgical overview. However, if the cholecystectomy is done first and the ERC fails, there is an increased risk of cystic stump leakage due to high pressure in case of an obstructive bile duct stone. In case the ERC will be performed first, a clamp can be placed on the distal duodenum prior to ERC to prevent further distribution of inflated gas through the bowel. When a re-ERCP is expected, for example, to remove or replace a stent, we suggest to directly place a PEG tube in the gastric remnant. The re-ERCP can be performed through this tube to prevent a re-laparoscopy and its associated adverse events.

The retrospective aspect and single-center setting are well-known limitations of our study. However, never before a series of this many LAERC was published.

We conclude that LAERC is an effective and relatively safe procedure for biliary diseases in patients with a RYGB. Whether this is the procedure of preference needs to be considered. Comparison with other methods is needed.
